# Openness in participation, assessment, and policy making upon issues of environment and environmental health: a review of literature and recent project results

**DOI:** 10.1186/1476-069X-10-58

**Published:** 2011-06-16

**Authors:** Mikko V Pohjola, Jouni T Tuomisto

**Affiliations:** 1 National Institute for Health and Welfare (THL), P.O.Box 95, FI-70701, Kuopio, Finland

## Abstract

Issues of environment and environmental health involve multiple interests regarding e.g. political, societal, economical, and public concerns represented by different kinds of organizations and individuals. Not surprisingly, stakeholder and public participation has become a major issue in environmental and environmental health policy and assessment. The need for participation has been discussed and reasoned by many, including environmental legislators around the world. In principle, participation is generally considered as desirable and the focus of most scholars and practitioners is on carrying out participation, and making participation more effective. In practice also doubts regarding the effectiveness and importance of participation exist among policy makers, assessors, and public, leading even to undermining participatory practices in policy making and assessment.

There are many possible purposes for participation, and different possible models of interaction between assessment and policy. A solid conceptual understanding of the interrelations between participation, assessment, and policy making is necessary in order to design and implement effective participatory practices. In this paper we ask, do current common conceptions of assessment, policy making and participation provide a sufficient framework for achieving effective participation? This question is addresses by reviewing the range of approaches to participation in assessment and policy making upon issues of environment and environmental health and some related insights from recent research projects, INTARESE and BENERIS.

Openness, considered e.g. in terms of a) scope of participation, b) access to information, c) scope of contribution, d) timing of openness, and e) impact of contribution, provides a new perspective to the relationships between participation, assessment and policy making. Participation, assessment, and policy making form an inherently intertwined complex with interrelated objectives and outcomes. Based on experiences from implementing openness, we suggest complete openness as the new default, deviation from which should be explicitly argued, in assessment and policy making upon issues of environment and environmental health. Openness does not undermine the existing participatory models and techniques, but provides conceptual means for their more effective application, and opens up avenues for developing new kinds of effective participatory practices that aim for societal development through collaborative creation of knowledge.

## Introduction

Stakeholder and public participation is undoubtedly one of the most central topics in contemporary discourse regarding environmental and environmental health policy and assessment. Environmental issues typically involve multiple interests regarding e.g. political, societal, economical, and public concerns and particularly in cases where they are known or perceived to relate either directly or indirectly to human health and well-being, the concerns often also become very personal. In such a setting of physical, chemical, biological, and societal complexity, it is widely accepted as important to include plural perspectives, particularly from the "affected parties", in the processes of policy making as well as the processes of producing information to guide and support policy making. As the idea of participation mainly builds on the theories and practices of democracy [1,2], this is particularly the case in the so called Western democracies, but increasingly also in countries not generally considered as democratic by their constitution, such as the People's Republic of China [3,4].

In addition to being founded on the principles of democracy, public participation is addressed in several intergovernmental agreements, e.g. the Principle 10 of the Rio Declaration [5], and the Aarhus Convention on Access to Information, Public Participation in Decision-Making and Access to Justice in Environmental Matters [6]. Also several laws on different levels of governance around the world, e.g. the EU Strategic Environmental Assessment Directive (2001/42/EC), the EU Public Participation Directive (2003/35/EC), The Law of the People's Republic of China on Environmental Impact Assessment [4], and the Finnish Environmental Impact Assessment (EIA) Act (468/94) and corresponding EIA Decree (713/2006), explicitly consider public participation and describe legal frameworks for its application. The legal requirements provide, however, only one perspective to participation. Importance of participation is also argued for example based on ethical, political, pragmatic, and epistemological [7] as well as substantive, normative, and instrumental reasons [1], and participation is seen to have the potential to deliver e.g. substantive, procedural, and contextual effects [8,9]. Participation in assessment and policy making upon issues of environment and environmental health has become commonplace.

This paper explores the following question: do current common conceptions of assessment, policy making and participation provide a sufficient framework for achieving effective participation? By effectiveness we mean influence on the outcomes, i.e. changes in values, attitudes, and behavior in the society (cf. [10]), of the processes that the participation relates to, e.g. participatory assessments or policy making.

Policy making is here understood as decision making upon issues of societal importance and assessments are considered as systematic science-based endeavors of producing information to support policy making. Public participation and stakeholder involvement are here seen as instances of the same issue which is mostly referred to as participation, meaning contributions from the parties, organizations or individuals that do not have formal roles as decision makers or experts in the assessment or policy processes in question.

These broad definitions allow inclusion of various types of participation, assessment, and policy making, practiced in and designed for several societal, institutional and geographical contexts by many different actors. For example, risk assessment, environmental impact assessment, and health impact assessment, whether practiced by consultants, federal agencies, or academic researchers, in Europe, USA, or China, are considered as just different manifestations of the fundamentally same process of science-based policy support. They are, however, clearly distinguishable from curiosity-driven research, ad hoc assessments, or assessments made to justify predetermined decisions. Here we focus on issues relevant to environment and environmental health, but the implications can be extended also to many other substantive contexts.

Answers to the question are sought for by discussing recent literature relevant to the question. That knowledge is complemented with some insights from recent research projects, INTARESE (Integrated Assessment of Health Risks of Environmental Stressors in Europe) [11] and BENERIS (Benefit-Risk Assessment of Food: an Iterative Value-of-Information Approach) [12].

INTARESE was an EU-funded research project running from 2005 to 2011, developing methodology and tools for integrated environmental health impact assessment (IEHIA), and testing them in case studies. BENERIS was also an EU-funded research project running from 2006 to 2009, developing a framework and tools for complicated benefit-risk situations, and applying them for analyzing benefits and risk of certain foods.

The review starts from purposes of participation and ends in consideration of openness. Overall it presents a new perspective to the relationships between participation, assessment, and policy making.

## Review

### Purpose of participation

The discourse on participation, involving both scholars and practitioners has primarily focused on implementation of participation while the multiple objectives and purposes of participation, particularly in relation to the objectives and purposes of the processes they relate to, have received much less attention [13]. This discourse has resulted for example in various guidance documents for stakeholder involvement [8,14,15], detailed presentation and discussion of various models for public participation, [16-21], and analysis of the applicability of participation techniques [22-24]. Although they are all important contributions to developing understanding about participation and its implementation, it is not always easy to identify how they link to the "outcome effectiveness of participatory processes in their societal context", as Newig [25] put it in developing his analytical framework for evaluating the impact of participation to improved environmental quality. Many means for public participation exist, but the ends they serve may not always be explicitly identified (for more on the theory of means-ends relationships, see e.g. [26]).

Despite the theoretical stance that participation is generally viewed as highly desirable and its benefits are often assumed to be obvious and substantial [13], the practices of policy making and assessment do not always represent this view. For instance, in a Finnish environmental permit case on a waste treatment activity the decision-maker, the permit applicant, as well as the stakeholders all questioned the meaningfulness of participation in the process, although in principle participation was seen as important by all [27]. The inconsistent utilization of public's contributions has also been seen as a general weakness in the Finnish environmental impact assessment system due to being strongly dependent on the developer's attitudes towards participation as well as the weak links between the assessment and related decision making processes [28]. This can be assumed representative of many other environmental impact assessment systems conducted under the EU Environmental Impact Assessment Directive (85/337/EEC) as well. Also in Canada the record of project-based environmental assessment in delivering on the promise of meaningful public participation has been identified as less than promising [29].

A major source of the problem with participation is that it has been more focused on process and access, rather than on outcomes [29]. It appears that the issue of effective participation needs to be considered in terms of the different possible purposes of both participation and assessment as well as their roles in the related policy making processes.

O'Faircheallaigh [13] has presented a nice characterization of ten specific purposes and activities, categorized under three broad purposes, for public participation in environmental impact assessment. The characterization is made in such a generic way, i.e. not bound to any specifics of contemporary environmental impact assessment practices, that we here assume it generalizable to all policy making and assessment regarding environmental and environmental health issues. According to O'Faircheallaigh [13] the three broad purposes for participation are:

• Obtain public input into decisions taken elsewhere

• Share decision making with public

• Alter distribution of power and structures of decision making

These categories roughly correspond to 1) participation influencing assessments and (potentially) their outputs, 2) participation influencing policy making and (potentially) policy decisions, and 3) participation as a means for influencing policy making from outside the existing institutional policy making structures. Within the broad purposes there can be several more specific sub-purposes, many of which are identified and discussed by O'Faircheallaigh [13], e.g. according to the kinds of expected, desired or allowed participant contributions. It is important to note that the purposes for participation are not exclusive, but can, and in fact often do, co-exist and interact. Advancing of different purposes of participation is strongly dependent on the attitudes towards participation among those who control the policy making and assessment processes, but also the types of interaction between assessment and policy making. Particularly this becomes apparent when attempting to advance several specific purposes of participation across categories, for example simultaneously filling information gaps with local knowledge, inviting public to decision making, and especially empowering marginalized groups (cf. [13]).

**Figure 1:**
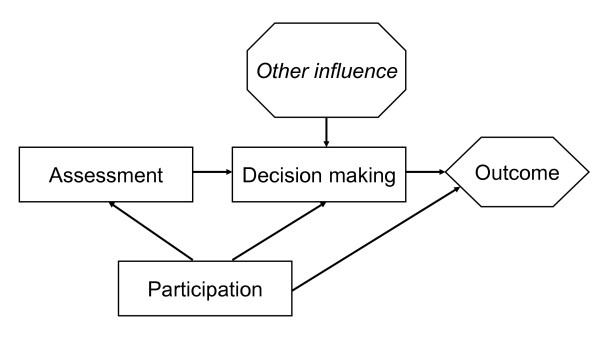
**Relationships between participation, assessment, policy making, and their outcomes**. Arrows depict alternative routes for potential influence from participation to outcomes.

The relationships between participation, assessment, policy making, and their outcomes are outlined in Figure 1 and discussed in following sections.

### Role of participation in assessment

There are multiple kinds of assessment that serve different purposes and address different kinds of questions, and thereby provide different contexts for participation. For example, Pope et al. [30] have differentiated between a) ex-post, project-based assessments (a typical kind for environmental impact assessment), b) ex-ante, objectives-led assessment (a typical kind for strategic environmental assessment), and c) (a more theoretical) assessment for sustainability. Briggs [31], on the other hand has differentiated between i) diagnostic assessment (does a problem exist, is policy action needed?), ii) prognostic assessment (implications of potential policy options, which option to choose?), and iii) summative assessments (effectiveness of existing policies). Assessment approaches may also be characterized according to their contexts of development and application as more regulatory or academic in their nature [32].

Many other classifications exist and new ones could be made, but what is important from the point of view of participation in assessments is the possible influence that is allowed for participation in different assessment settings. For example, does the assessment structure allow for rethinking a project at the time the public is engaged in a project-based environmental assessment (cf. [29])?, can the stakeholders influence the choice of policy options to be considered in a prognostic integrated environmental health impact assessment (cf. [31])?, or does a down-stream user of a chemical product have any other role besides providing assessors with information on specific chemical use contexts in a REACH (Registration, Evaluation, Authorisation and Restriction of Chemical Substances (EU)) chemical safety assessment [33]? The framing of an assessment approach can be a significant constraining factor for potential effectivness of participation.

Quite often stakeholder involvement and public participation are seen as specific steps or stages in the assessment process (e.g. [31,34,35], Finnish EIA Act (468/94)), which may limit the possible influence of participation to only certain questions topical at that particular stage. Also according to the study on the state-of-the-art in benefit-risk analysis conducted in the BEPRARIBEAN (Best Practices for Risk-Benefit Analysis of Foods) project [36], the commonly applied, contemporarily well established approaches to environmental health assessment treat stakeholder involvement and public participation rather as an add-on, often brought about by legal requirements, than as an essential aspect of assessment or decision making processes [32].

### Role of participation in policy making

On the other hand, in many aspects the level of influence that participation in assessments can have is not directly in control of the assessors. For example, in the aforementioned Finnish environmental impact assessment system, where participation is legally enforced as a part of the assessment process, the decision making structures outside the assessment may induce that certain aspects of assessment results, e.g. in particular public concerns regarding social impacts, cannot be given weight in the decision making [28]. Also the Finnish land use planning system, in which there are legal requirements for impact assessment including public participation, treats planning (zoning) and development as separate processes, which means that the details of planned development, issues of great public interest, are outside the scope of assessment and stakeholder involvement [37]. Both of these examples describe national implementations of EU directives, and are thereby somewhat representative of the whole target area of the corresponding EU legislation.

The influence of assessment, participation, as well as all other potential inputs to policy making is much determined by the setting in which policy decisions are being made. As an example from another kind of societal context, the Chinese authorities may welcome public participation if it improves the quality of information available to government decision makers, but may not at all be willing to give public the power to contribute to and influence decision making by participating in the formulation of a proposal, the whole assessment process, the implementation, and the evaluation of a proposal [4,13].

### Indirect participatory influence

If no satisfactory roles are provided for public, or even expert, input directly in decision making or indirectly through assessment, alternative options for influencing policy need to be looked for outside the institutionalized decision making structures, as pointed out also by O'Faircheallaigh [13]. In fact, quite many, particularly the more academic, assessment approaches, although explicitly aiming to support policy making, do not explicitly describe their linkages to any particular specific policy uses [32]. This may be interpreted indicative of their, more or less implicit, intentions to activate also other channels than only direct influence to policy making. An alternative way to advance the societal purposes of assessments is e.g. to promote social learning among public officials, market players, and citizens. Also in the recent evaluation of the existing EIA legislation in Finland the indirect influence of the information and knowledge obtained in the participatory assessment, which does not directly serve the formal sectoral permit decision processes related to the assessed project, was interpreted as an important aspect of the Finnish EIA system by contributing to the general awareness among the society upon the environmental and health impacts of on-going developments [38].

### Assessment-policy interaction

As has been pointed out above, the interaction between assessment and policy making can be crucial for effective participation. Another question then is what influences the assessment results, potentially influenced by participation, have in the related decision making processes. Although often quite credulously assumed by assessors and assessment scholars that assessments have significant impacts to the decision making processes they aim to serve (cf. [13]), few approaches to assessment actually even explicitly consider assessment performance in terms of the outcomes of using the assessment results in their intended contexts of use [32,39]. Concerns have also been expressed that the emphasis in environmental impact assessment has been more on process and procedure, rather than on purpose and effects [40,41].

Assessment-policy, as well as related science-policy and research-practice, relationships have recently been subjects of intense discussion and several characterizations of the interfaces or boundaries in between them have been presented from different viewpoints. For example Sterk et al. [42] have characterized five boundary arrangements of varying levels of engagement between science and policy, van Kerkhoff and Lebel [43] have presented six categories of relationships between research-based knowledge and action, a continuum of increasing engagement and power sharing, and Cashmore [40] has described a spectrum of five models representing varying conceptions of the role of science, and participation, in environmental impact assessment. In addition, the relationships have been considered in multiple other discourses, e.g. on trans- and interdisciplinary research [44-47], regulatory science [48], Integration and implementation sciences [49], post-normal science [50], integrated research [51], informing science [52], knowledge brokerage [53,54], science integrators [55], boundary organizations, objects and systems [56-58], science-policy interfaces [59], participatory integrated assessment [60,61], environmental health assessment [32], making use of science in policy [62-64], adaptive governance [65-67], policy integration [68], policy practice [69], and policy analysis [70].

Although the viewpoints, bases and contexts in the above mentioned discourses vary, in aggregate they seem to be pointing to the direction of increased openness. According to our interpretations in the context of this paper, of the main lessons from these discourses regarding assessment-policy interaction and participation are as follows:

• The traditional model of disengaged scientific assessment and policy making is increasingly considered both by policy makers and researchers as inadequate to address existing policy needs sufficiently

• There is a need for more pragmatic needs-oriented question setting in assessments

• Deeper engagement between assessment and policy making is essential for policy effectiveness

• Stakeholder and public participation is essential for relevance both in assessment and policy making

• Values are an important aspect of the needed knowledge input for both assessment and policy making.

This broad gradual movement could be characterized as a shifting of both assessment and participation from the lower degrees of involvement, e.g. informing or information collection, towards the higher degrees of involvement, e.g. co-deciding, delegated power, joint planning, or partnering, in relation to policy making (cf. [8,15,20,40]). The shift can also be identified e.g. by observing the development in the perspectives to the relationship between risk assessment and risk management adopted in the publications of the NRC (National Research Council (USA)): from strict disengagement in the so called Red Book [71] to binding through deliberative characterization in the so called Orange Book [72], and on to an intertwined process of risk-based decision making in the recent so called Silver Book [73]. Also the role of stakeholder involvement has grown alongside the development of assessment-policy interaction.

Participation, assessment, and policy making are becoming to be perceived as an intertwined complex that needs to be considered as a whole, not as separate independent entities. The question of effective participation is thus meaningful only in the broader context that also concerns the purposes and effects of policy making and the processes of producing the knowledge that it is based on. However, as has been pointed out above, the common current practices of participation, assessment, and policy making are not necessarily always in line with the latest discourses in the literature.

### Dimensions of openness

One obstacle for effectively addressing the issue of effective participation may be the concept of participation itself. As long as the discourse focuses on participation, one is easily misled to considering it as an independent entity with purposes, goals and values in itself, without explicitly relating it to the broader context of the processes whose purposes it is intended to serve. The conceptual framework we call the dimensions of openness attempts to overcome this obstacle by considering the issue of effective participation in terms of openness in the processes of assessment and decision making. The framework was developed as a part of the assessment methodology development in the INTARESE project, and it is intended as guidance for designing and managing participatory assessment and decision making practices. In the project, the development work was originally motivated by a notion of a simultaneous need to improve effectiveness of assessments in environmental health policy making as well as to improve effectiveness and meaningfulness of stakeholder involvement in environmental health assessments.

As the name implies, the framework consists of five essential dimensions of openness in assessment and decision making, or more generally, creation and use of collective knowledge. The dimensions of openness does not attempt to provide an exhaustive and mutually exclusive list of all aspects of openness, but to explicate and emphasize those that are seen as the most essential ones in the context of environment and health assessment and policy. The five dimensions of openness are:

• **Scope of participation**, referring to who are allowed to participate in the process.

• **Access to information**, referring to what information regarding the issue at hand is made available to participants.

• **Timing of openness**, referring to when participants are invited or allowed to participate.

• **Scope of contribution**, referring to which aspects of the issue at hand participants are invited or allowed to contribute to.

• **Impact of contribution**, referring to what extent are participant contributions allowed to have influence on the outcomes, i.e. how much weight is given to participant contributions.

The dimensions of openness compile the main issues of participation in one solid framework. In the framework, the more commonly addressed questions of access (to process and to information) and timing of participation are complemented with less commonly addressed questions of extent and influence of participation on the outcomes of the process. The five dimensions can be considered as the determinants of the possibilities and limitations provided by the context for the effectiveness of participation. As such, the framework explicates the aspects of openness that need to be taken account of in order to match the processes and procedures of collective knowledge creation and use, e.g. environmental health assessment and related policy making, with their aims and purposes. Thereby it also provides a means for identifying the relationships between participation, assessment, and decision making.

The framework bears resemblance e.g. to the criteria for evaluating implementation of the Aarhus Convention principles by Hartley and Wood [23], the categories to distinguish a discrete set of public and stakeholder engagement options by Burgess and Clark [74], and particularly the seven categories of principles of public participation by Webler and Tuler [75]. However, whereas they were constructed for the use of evaluating or describing existing participatory practices or designs, the dimensions of openness framework is explicitly and particularly intended to be used as a checklist type guidance to support design and management of participatory assessment and decision making processes.

The perspective adopted in the framework can be characterized as contentual because it primarily focuses on the issue in consideration and describing the prerequisites to influencing it, instead of being confined to only considering techniques and manoeuvres to execute participation events. Thereby it helps in participatory assessment and decision making processes to achieve their objectives, and on the other hand in providing possibilities for meaningful and effective participation. The framework does not, however, tell how participation should be arranged, but rests on the existing and continually developing knowledge base on participatory models and techniques. Although a contentual perspective to participation, dimensions of openness does not contradict with the procedural perspectives to participation, but rather provides a backdrop for their effective application.

The contentual perspective makes the framework applicable in design and management of both assessment and policy making processes. Although assessment and decision making may appear as very different kinds of processes, the choice of point of view is actually only a question of adjusting the scope of application of the framework; whether decision makers are included in an assessment or not? After all, assessment and related decision making should ideally only be alternative perspectives to the same issue, the former emphasizing the development of knowledge, the latter emphasizing the use of knowledge. Within the contentual perspective, everyone, including also e.g. authorities, project managers, and experts, not only public, stakeholders, NGO's (non-governmental organizations) etc., are considered as participants to development and implementation of knowledge. They are all considered as, at least potentially, relevant contributors to either creating knowledge or deciding about an issue of interest. The different kinds of participants naturally take different roles according to their interests, capabilities, professions, as well as formal and legal positions in relation to the issue.

The degree of openness can be managed in terms of the dimensions of openness according to specific purposes and goals. The situational, contextual, and practical issues, for example legal requirements, public perceptions, available resources, time constraints, complexity of the case, confidentiality etc. also need to be taken account of in deciding upon suitable degree of openness. The degree of openness can be adjusted separately for different groups of participants, or even on an individual basis, and varying from a case to another, as needed. The overall openness of the process can be considered as a function of all five dimensions across all roles, although it should be noted that the dimensions are not independent, but rather interrelated.

For example, the first dimension, scope of contribution, determines the participant groups among which questions regarding e.g. access to information or scope of contribution are only even relevant. In addition, while all dimensions contribute to the overall openness, it is the fifth dimension, the impact of contribution, which ultimately determines the effect on the outcome. Accordingly, it is recommended that aspects of openness in assessment and decision making processes are considered step-by-step, following the order as presented above.

The greatest power of the framework is that it puts the issue at hand in focus and does not build on any preconceptions about possible or acceptable inputs to its development. It allows to first ask what are the inputs needed to develop the issue to achieve its purpose, and then consider the arrangement for its realization, without being preconfined to existing conventions and institutions of participation, assessment and decision making, which, as argued above, are in many cases known to be inadequate. The framework i) provides a context for evaluation and constructive criticism of existing conventions and institutions, ii) facilitates innovative application of existing means for participatory processes within and alongside the existing conventions and institutions, and iii) promotes development of new means, conventions and institutions for participatory practice. Thinking in terms of openness provides a new perspective to participation in assessment and policy making.

### Implementation of openness

The first version of the dimensions of openness framework was developed already in the early phases of the INTARESE project. At the same time also an alternative, procedural, assessment approach was developed within the project, eventually leading to the formulation of the IEHIA method [31]. Although in retrospect it can be seen that the dimensions of openness framework and the IEHIA method are complementary rather than fundamentally contradictory, some practical difficulties in merging these views led into their development side by side rather than together within the project. Consequently, the dimensions of openness framework was eventually taken for further development and application also outside the project. In practice, this meant that the authors, also the initiators of the framework development, began to develop and apply the dimensions of openness framework in their work for developing a new, more holistic approach for environmental health risk analysis within one of the partnering institutions, the National Institute for Health and Welfare (THL) from Finland. The approach was first known as Pyrkilö (originating from the Finnish word pyrkiä, to strive for), and later as open risk assessment [76]. Eventually the method became named open assessment and the web-workspace for conducting open assessments became named Opasnet [32,77-79]. A major part of the early development of the Opasnet web-workspace was particularly carried out in the BENERIS project.

**Figure 2:**
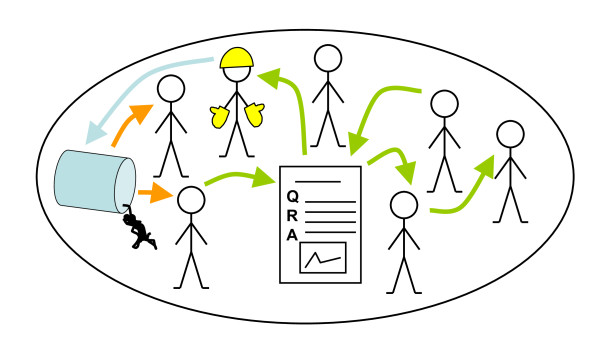
**Open assessment as a collaborative social knowledge process**. The strawmen depict the members of a society. The paper sheet depicts an assessment in Opasnet (Q = question, R = rationale, A = answer). Yellow arrows depict observation (here of an undesired event, a toxic liquid spill). Green arrows depict information flow (from members of society to an assessment in Opasnet, from the assessment to members of society, or directly between members of society). Blue arrow depicts knowledge-based action (correctly taking care of the spill).

The method development work took account of several of the aspects of policy making, assessment, and participation discussed above, and was influenced by the research results and experiences e.g. on collective knowledge creation in fields of education, psychology, and philosophy [80], computer-supported collaborative learning [81,82], mass collaboration [83] as well as crowd-sourcing [84,85]. Application of the framework in this setting led into a somewhat extreme interpretation of participatory practice in the context of environmental health: the assessments should be made completely open by default and limitations in degree of any dimension of openness should be done only based on cogent and explicitly argued reasons! The framework also illuminated that assessments, the knowledge creation processes, need to be deeply intertwined with the decision making processes, the knowledge use, if they seriously attempt to achieve their purposes of influencing policy. This makes decision makers a particularly essential kind of active assessment participants. These ideas, quite contrary to the currently common conceptions of assessment and participation, became two of the fundamental principles guiding open assessment method and Opasnet web-workspace development. The idea of open assessment is illustrated in Figure 2.

The unconventionality of the principle of complete openness, and how it relates to the interrelations between participation, assessment, and decision making, is illustrated by the comparison, in terms of the dimensions of openness, of five assessment approaches relevant in the field of environment and environmental health in table 1. The comparison also demonstrates the application of the framework in evaluating existing practices. For the sake of clarity the focus of comparison is here on "external participation" in the assessment processes, e.g. by decision makers, stakeholders or public. The possible differences in the roles of the expert assessors nominated for the assessment task are thus not explicitly considered. The five assessment approaches included in the comparison are 1) Open assessment [32,76-79] (State of the art in benefit-risk analysis: Environmental health. Unpublished manuscript developed in the BEPRARIBEAN project), IEHIA [31], YVA - the Finnish environmental impact assessment system (Finnish EIA Act 468/94) [28], the so called "Red Book" risk assessment [71], and its recent update, the "Silver Book" risk-based decision making framework [73].

**Table 1:** Perspectives to openness in "external participation" for five example assessment approaches considered according to the dimensions of openness framework.

Dimension/Approach	Scope of participation	Access to information	Timing of openness	Scope of contribution	Impact of contribution
Open assessment	Everyone, e.g. decision makers, NGO's, citizens, external experts, allowed to participate. User participation particularly important.	All information should be made available to all participants.	Continuous.	All aspects of the issue can be addressed by everyone.	Based on relevance and reasoning, not source. All relevant contributions must be taken into account. Conclusions from collaboration intended to turn into action through collective knowledge creation among participants in a shared web-workspace.

IEHIA	Specified users (e.g. policy makers), and stakeholders (preferably by proxy) invited to participate.	?	User and stakeholder participation during issue framing, design and appraisal phases (not during execution phase).	Users and stakeholders can participate in scoping and design of assessment and interpretation of results.	Participant views influence the construction of the assessment framework. Appraisal phase discourse regarding the assessment results, their implications for action, and their linkage to the goals defined in issue framing assumed to ensure that those involved accept the outcomes.

YVA	Public, liaison authority (e.g. regional environmental center), other authorities.	Assessment plan and assessment report provided to the public by the project developer. Liaison authority also has access to information regarding e.g. other plans, projects and operations relevant to the project.	Participation in two phases. Public hearing periods, possible authority statements regarding both assessment plan and assessment report. Liaison authority gives its statements after the public and the other authority statements.	Any public representative can give any statements, and the liaison authority may ask specific statements from other authorities in both phases. Liaison authority gives an overall statement on both the assessment plan and the assessment report.	Public statements filed along with the liaison authority statements. Ultimately up to the project developers and the decision makers to decide if and how public statements are taken account of in project design or decision making. The liaison authority, also taking account of public and other authority statements, can also demand e.g. certain issues to be considered in the assessment or other additional information to be provided by the project developer.

Red Book	N/A (Assessment for nominated scientific experts only).	N/A	N/A	N/A	Assessment results provided for decision makers and intended to be taken into account, alongside options evaluation, in decision making and action by federal agencies.

Silver Book	Decision-makers, technical specialists, and other stakeholders.	Formal provisions for internal and external stakeholders at all stages.	At all stages: problem formulation and scoping, planning and conduct of risk assessment, and risk management.	Problem formulation and scoping, confirmation of utility of risk assessment, and risk management.	Stakeholders as active participants. However, participation should in no way compromise the technical assessment of risk, which is carried out under its own standards and guidelines.

? = could not be determined based on the information source, N/A = not applicable.

In addition to describing varying degrees of openness, the comparison illustrates striking differences in how the approaches see the interrelations between participation, assessment, and decision making. Open assessment and the Red Book risk assessment represent the two extremes. Whereas the former sees participation, assessment, and policy making as aspects of the same collaborative process, the latter does not even consider participation and explicitly recognizes interaction between assessment and the external world only in distribution of assessment results to decision making. The three other examples, IEHIA, YVA, and Silver Book fall in between these extremes by allowing some degrees of openness, although in somewhat different ways, and identifying linkages between participation, assessment, and decision making. However, as was mentioned earlier, the linkage from YVA assessments, and participation organized within them, is known to be weak. Also for IEHIA, the description of the relationship between assessment and decision making remains quite implicit. The Silver Book perspective makes a radical update to the Red Book perspective, yet it still retains the risk assessment as a fundamentally independent and exclusive expert process.

The scrutiny of openness according to the dimensions of openness framework can, as exemplified above, reveal interesting aspects of participation, assessment, and policy making practices, and their potential to deliver what they intend to. Although in many discourses participation seems to be assumed to have "value in itself", or plainly seen to "belong to democracy", from a contentual point of view this kind of reasoning misses the main point of openness. Openness is not an end in itself, but rather a means for advancing societal development through creation and use of broadly distributed collective knowledge. Openness calls into question the assumptions behind the institutional practices that we have accustomed ourselves to take for granted.

For example, complete openness, as adopted in open assessment, actually applies an inverse interpretation of the dimensions of openness, i.e. who should not be allowed to participate, what information should not be made available to participants etc. From this perspective it often becomes difficult to argue e.g. for exclusion of any specific groups or individuals from assessments, or withholding important information, especially if also the arguments are exposed to open critique. Particularly this is the case in the context of environment and environmental health, where the issues addressed are often relevant to virtually everyone and every organization or individual is a potential source of relevant contributions. As an example, issues regarding global climate change involve a nearly infinite amount of actors in different roles e.g. as contributors to climate change, its mitigation and adaptation, or parties affected by impacts of climate change or its mitigation and adaptation actions.

Openness necessarily also requires a more inclusive view to assessment than what the conventional conceptions of assessment provide. Assessment should not only be confined to mean the expert-driven, natural science influenced, fact-based, sometimes strictly quantitative, so called scientific assessments aiming to find objective answers. It should also extend to explicit inclusion of values and all kinds of knowledge from all sources, qualitative treatment of information, and creation of contextual, situational, and pragmatic knowledge among assessment participants. Such a conception of assessment actually ideally also includes decision making, and other possible uses of knowledge created by assessment. Although confronted with "scientific assessment" above, the open conception of assessment is actually not any less scientific. After all, the heart of science is in development of shared belief systems based on open critique, evidence, interpretation, and argumentation, which necessitates openness. Creation of new fora for scientific discourse, and invitation of new participants and new topics to enter these fora does not in itself guarantee scientifically valid outputs, but it provides possibilities for overcoming the identified limitations to effectiveness in policy making, assessment, and participation in the conventional approaches that build on exclusivity and disengagement rather than openness.

### Challenges of openness

Openness definitely also brings about significant challenges in terms of e.g. manageability of broad participation, information quality control, prevention from intentional bias or promotion of vested interests, protection from vandalism, cost and time expenditure, etc. (cf. [14]), but we claim that these problems are rather practical than fundamental in their nature. Nevertheless, they are real challenges to practical implementation of openness. Methods and tools to help overcome many of these challenges already exist or are being developed (see e.g. the references regarding influences to open assessment development above) and some of the problems may even be solved by increased openness itself.

For instance, within the conventional frameworks openness is often assimilated with time-consuming appeals, endless disputes, and costly stakeholder involvement actions. However, well designed and executed open assessment or policy processes can also mean high acceptability of outcomes, rapid solutions to well-defined problems through web-based collaboration, and improved societal impacts.

Professional assessors and policy makers may, and often do, fear losing their power in open processes, and it is true that openness does affect their roles. The experts in assessment or decision making should see themselves not as obtaining inputs to their own private assessment or policy processes, but rather as feeding the open collaborative processes of assessment and policy making (as in Figure 2) with their expertise.

However, the law seldom requires very high degrees of openness, and legislation regarding participation is usually built on the conventional frameworks. Thereby the conventions will probably have to change first, before more support for the shift towards openness can be expected from legislation regarding environmental and environmental health assessment and policy making. Perhaps in the end the greatest challenge lies in the scientists', assessors' and decision makers' attitudes towards openness, and the internal resistance to change contemporary research, assessment and decision making practices more open.

## Conclusions

In conclusion, based on the review of literature and insights from recent research projects, we state that:

1. Inclusion of stakeholders and public to participate in assessments and policy making upon issues of environment and environmental health is an issue of both great interest and importance.

2. The discourses on both assessments and participation in the contexts of environment and environmental health have been too much focused on processes and procedures, and too little attention has been given to their purposes and outcome effectiveness in policy making.

3. Consideration of effective participation is meaningful only in the context of purposes and effects of the assessment and policy making processes that participation relates to.

4. The dimensions of openness framework provides a conceptual means for identifying and managing the interrelations between the purposes and outcomes of participation, assessment, and policy making, and thereby also for effective application of existing participatory models and techniques.

5. The dimensions of openness framework also provides a context for evaluation and constructive criticism of contemporary conventions and institutions of participation, assessment, and policy making, and a basis for developing new conventions and institutions.

6. From a contentual point of view, it can be argued that participation, assessment, and policy making upon environmental and environmental health issues should be considered as completely open rather than exclusive processes by default.

7. Openness should not, however, be considered as an end in itself, but rather a means for advancing societal development through creation and use of broadly distributed collective knowledge upon issues of great societal relevance.

8. Openness brings about challenges, but they are mostly practical, rather than fundamental in their nature.

## Abbreviations

THL: National Institute for Health and Welfare (Finland); INTARESE: Integrated Assessment of Health Risks of Environmental Stressors in Europe (EU-funded research project); BENERIS: Benefit-Risk Assessment of Food: an Iterative Value-of-Information Approach (EU-funded research project); EU: European Union; EC: European Community; EIA: Environmental impact assessment; USA: United States of America; IEHIA: Integrated environmental health impact assessment; EEC: European Economic Community; BEPRARIBEAN: Best Practices for Risk-Benefit Analysis of Foods; REACH: Registration, Evaluation, Authorisation and Restriction of Chemical Substances (EU); NRC: National Research Council (USA); NGO: Non-governmental organization; YVA: Environmental impact assessment (acronym in Finnish); UNEP: United Nations Environment Programme; UNECE: United Nations Economic Commission for Europe; ECLAC: Economic Commission for Latin America & the Caribbean; OECD/NEA: Organisation for Economic Co-operation and Development/Nuclear Energy Agency; IIED: International Institute for Environment and Development; MNP: Netherlands Environmental Assessment Agency (current acronym for the agency is PBL); NIBR: Norwegian Institute for Urban and Regional Research; ECHA: European Chemical Agency; ITRC: The Interstate Technology & Regulatory Council.

## Competing interests

The authors declare that they have no competing interests.

## Authors' contributions

The review of literature and preparation of the paper have mainly been responsibilities of MVP. JTT has also contributed to the analysis of the literature in the light of the presented framework and drawing conclusions based on the analysis. The revisions, as requested by reviewers, were mainly made by MVP. MVP and JTT are the two main figures behind the development of the dimensions of openness framework, open assessment method and the Opasnet web-workspace. All authors read and approved the final manuscript.
